# TRPV4 mediates aminoglycoside trafficking and ototoxicity without compromising antimicrobial efficacy

**DOI:** 10.1038/s41420-026-03132-9

**Published:** 2026-04-25

**Authors:** Lingshuai Kong, Takaomi Kurioka, Sachiyo Mogi, Yoshihiro Nitta, Kengo Yamamoto, Taku Yamashita

**Affiliations:** 1 Department of Otorhinolaryngology, Head and Neck Surgery, Kitasato University, Sagamihara, Japan; 2 Department of Otolaryngology, Shanxi Children’s Hospital, Taiyuan, China; 3 School of Allied Health Science, Kitasato University, Sagamihara, Japan

**Keywords:** Cochlea, Neurodegeneration

## Abstract

Aminoglycoside (AG) antibiotics remain essential for treating life-threatening infections; however, their clinical use is limited by irreversible ototoxicity. The mechanisms of AG entry into the cochlea and its role in cochlear degeneration remain unclear. This study identified the transient receptor potential vanilloid 4 (TRPV4) channel as a key mediator of AG trafficking and ototoxicity. In an AG-induced ototoxicity mouse model, TRPV4 pharmacological inhibition reduced cochlear AG accumulation, preserving hearing, hair cell (HC) survival, and cochlear synaptic integrity. Conversely, TRPV4 pharmacological activation accelerated cochlear AG influx, exacerbating AG-induced ototoxicity. Cochlear explant experiments showed that TRPV4 agonists and antagonists modulated Texas Red-labeled gentamicin uptake and HC survival, suggesting that TRPV4 directly contributes to the regulation of HC permeability and survival in the explant preparation. Notably, TRPV4 modulation did not compromise AG antimicrobial activity in bacterial-killing assays, uncoupling therapeutic efficacy from ototoxicity. Collectively, these findings identify TRPV4 as a molecular gateway for AG trafficking into cochleae and demonstrate that its inhibition offers a strategy to protect hearing while maintaining antimicrobial potency. Thus, targeting TRPV4 may provide a clinically translatable approach to prevent AG-induced ototoxicity without undermining their life-saving benefits.

## Introduction

Aminoglycoside (AG) antibiotics are essential for treating life-threatening bacterial infections, particularly in low- and middle-income countries where frontline therapies are used [1, 2]. Despite their utility, dose-dependent ototoxicity causes permanent sensorineural hearing loss in 10–20% of patients, with children and older individuals being the most vulnerable [3, 4]. Hearing loss imposes burdens on communication, education, and socioeconomic outcomes, making it a major health concern [5]. The cellular basis of AG-induced hearing loss (AGHL) has been studied for decades. AGs accumulate in cochlear hair cells (HCs), spiral ganglion neurons (SGNs), and the stria vascularis (SV), inducing reactive oxygen species (ROS) production, mitochondrial dysfunction, calcium dysregulation, and apoptosis [6, 7]. While downstream pathogenic cascades are well characterized, the upstream molecular mechanisms mediating AG entry into the cochlea remain poorly defined, limiting clinical translation [5]. This necessitates strategies that regulate AG trafficking into the cochleae without impairing systemic antibacterial action.

Transient receptor potential (TRP) channels regulate cochlear ion transport and drug permeability [6]. Transient receptor potential vanilloid 4 (TRPV4) is a calcium-permeable nonselective cation channel expressed in the cochlea, including HCs, SGNs, and SV [1]. TRPV4 participates in mechanotransduction (MET), osmosensation, and barrier permeability, and its activation promotes calcium influx and apoptosis in conditions such as ischemia-reperfusion injury and neurodegeneration [2]. TRPV4 activity is linked to noise-induced and age-related auditory decline [8], suggesting a role in cochlear vulnerability. Despite these associations, TRPV4’s role in AG trafficking and ototoxic injury remains undefined. This study investigated TRPV4 as a gateway for AG entry into the cochlea. Pharmacological approaches showed that TRPV4 mediates AG trafficking and ototoxicity. TRPV4 inhibition preserved hearing, HC survival, and synaptic integrity without impairing antimicrobial efficacy. These findings identify TRPV4 as a key regulator of AGHL and highlight its inhibition as a translatable strategy to separate antibacterial benefits from ototoxicity.

## Results

### Mouse model of aminoglycoside-induced hearing loss (AGHL)

**Fig. 1:**
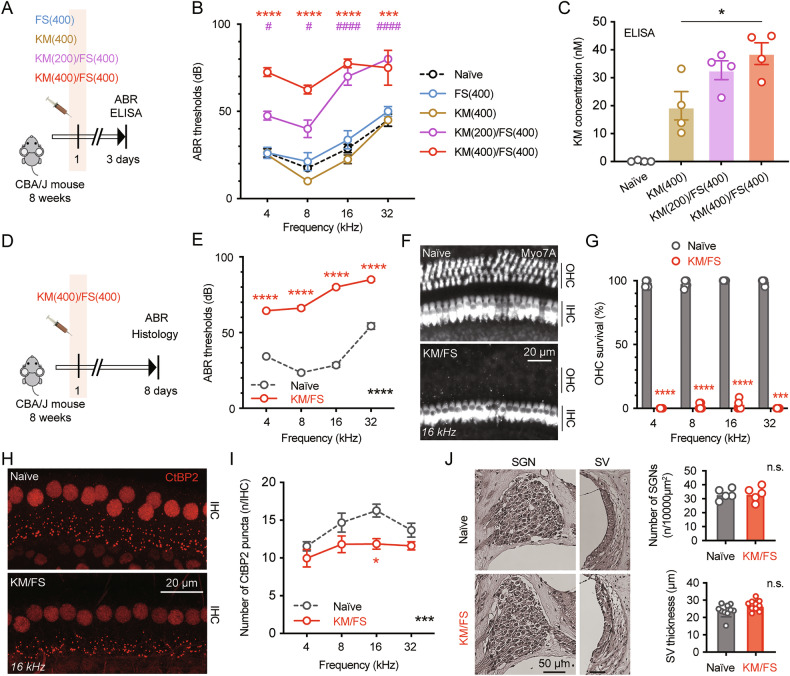
AG-induced ototoxicity animal model. **A** Experimental protocol for evaluating KM-induced ototoxicity. **B** ABR thresholds measured 3 days after administration of different KM/FS doses. KM(200)/FS(400) and KM(400)/FS(400) mice showed significant elevations in hearing thresholds compared with naïve mice. *n* = 4 per animal group. **C** ELISA quantified cochlear KM concentrations 3 days after administration of different KM/FS doses. The KM concentration in KM(400)/FS(400) was significantly higher than that in KM(400). *n* = 4 per animal group. **D** Experimental protocol for evaluating ABR thresholds and cochlear pathology following KM(400)/FS(400) administration. **E** ABR thresholds 1 week after KM/FS administration. KM/FS mice showed significantly higher ABR thresholds than naïve mice. *n* = 5 per animal group. **F** Representative immunostaining of HCs with Myo7A, 1 week after KM/FS administration. Severe OHC loss was apparent in KM/FS mice. **G** Quantification of OHC survival 1 week after KM/FS administration. The OHC survival rate in KM/FS mice was significantly lower than that in naïve mice. *n* = 5 per animal group. **H** Representative immunostaining of synaptic ribbons with CtBP2 1 week after KM/FS administration. KM/FS mice showed fewer cochlear ribbon synapses than naïve mice. **I** Quantification of CtBP2-positive synaptic ribbon 1 week after KM/FS administration. The number of synaptic puncta significantly decreased in KM/FS mice. *n* = 4 per animal group. **J** Representative hematoxylin–eosin staining of the cochlea and quantification of SGN survival and SV thickness at the cochlear middle turn 1 week after KM/FS administration. KM/FS mice showed no significant changes in SV and SGN compared with naïve mice. *n* = 5 per group for SGN assessment and *n* = 10 per group for SV assessment. Asterisks indicate statistically significant differences. *, ^#^*p* < 0.05, ***, ^###^*p* < 0.001, ****, ^####^*p* < 0.0001. ABR auditory brainstem response, FS furosemide, IHC inner hair cell, KM kanamycin, OHC outer hair cell, SGN spiral ganglion neuron, SV stria vascularis.

Initially, a murine model of AGHL was established via systemic kanamycin (KM) and furosemide (FS) administration at varying concentrations, evaluating auditory function and intracochlear KM concentrations using an enzyme-linked immunosorbent assay (ELISA) (Fig. 1A). A single intraperitoneal injection of KM at 400 mg/kg (KM(400)), FS at 400 mg/kg (FS(400)), or normal saline (naïve) did not elevate auditory brainstem response (ABR) thresholds 3 days post-administration (two-way analysis of variance (ANOVA), KM(400) vs. naïve, *p* = 0.69, FS(400) vs. naïve, *p* = 0.59, Fig. 1B), consistent with previous reports of adult mouse resistance to AG [9, 10]. However, the combination of 200 mg/kg KM with 400 mg/kg FS (KM(200)/FS(400)) or 400 mg/kg KM with 400 mg/kg FS (KM(400)/FS(400)) significantly increased ABR thresholds (two-way ANOVA, KM(200)/FS(400) vs. naïve, *p* < 0.0001; KM(400)/FS(400) vs. naïve, *p* < 0.0001), indicating that combined KM/FS treatment provides an effective approach to overcome resistance to ototoxicity. To investigate the mechanisms underlying the enhancement of ototoxicity by FS, intracochlear KM concentrations were measured 2 days following injection using ELISA. KM concentrations in whole cochleae increased in the group co-administered FS, especially KM(400)/FS(400), demonstrating a marked elevation compared with KM(400) alone (one-way ANOVA, KM(400)/FS(400) vs. KM(400), *p* = 0.01, Fig. 1C). These findings align with previous studies, indicating that FS, when co-administered with KM, exacerbates KM-induced ototoxicity [11].

As a single administration of KM combined with FS induced significant hearing loss in the mouse model, KM(400)/FS(400) was selected as the KM-induced ototoxicity mouse model for subsequent experiments. One week after a single intraperitoneal administration of KM(400)/FS(400), cochlear electrophysiological and histological analyses were performed (Fig. 1D). ABR measurements revealed significantly elevated hearing thresholds across all measured frequencies in KM/FS-treated mice (two-way ANOVA; KM/FS vs. naïve, *p* < 0.0001; Fig. 1E). For histological assessment of the inner ear, surviving cochlear HCs were initially evaluated via immunohistochemical staining for myosin7A (Myo7A), a marker of HCs. In normal mice, three rows of outer HCs (OHCs) and one row of inner HCs (IHCs) were aligned, whereas in the KM/FS group, marked OHC loss was observed, with IHCs remaining largely viable (Fig. 1F). Quantification of surviving HCs indicated a significant reduction in OHCs in KM/FS mice across all frequency regions examined, consistent with the significant elevation in ABR thresholds (two-way ANOVA, KM/FS vs. naïve, *p* < 0.0001, Fig. 1G). To further assess cochlear ribbon synapses at IHC–cochlear nerve junctions, which are primary targets of ototoxic stimuli [12], ribbon synapses were evaluated by CtBP2 immunostaining (Fig. 1H). A significant reduction in the number of ribbon synapses was observed in the KM/FS group (two-way ANOVA, KM/FS vs. naïve, *p* = 0.0003; Fig. 1I). Furthermore, cross-sectional evaluation revealed no discernible histological changes in either SV thickness or SGN survival in the cochlear middle turn 1 week after KM/FS administration (Mann–Whitney, SV thickness, *p* = 0.09; SGNs, *p* > 0.99, Fig. 1J). These findings suggest that OHCs and cochlear ribbon synapses are the primary targets of cochlear damage following KM/FS treatment.

### Intracochlear expression of TRPV4 and its change in expression after KM administration

**Fig. 2:**
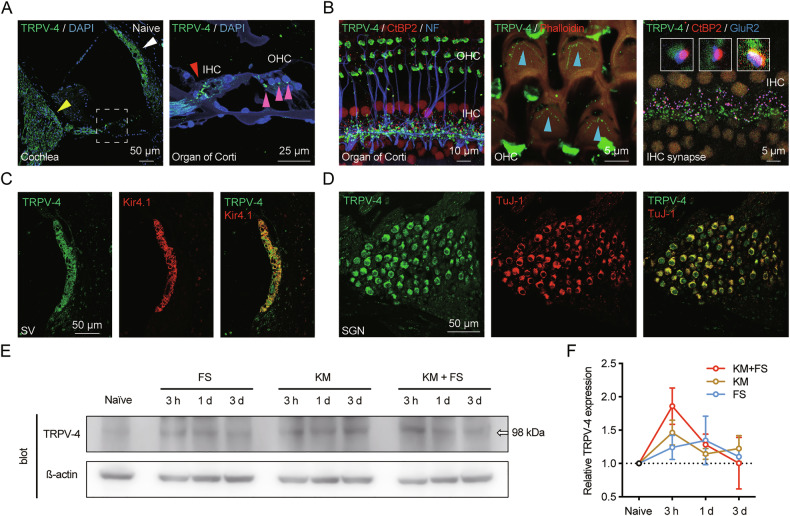
TRPV4 expression in the mouse cochlea. **A** Representative micrographs of cochlear sections immunostained for TRPV4 in naïve mice. TRPV4 expression was observed in the SV (white arrowhead), SGN (yellow arrowhead), and organ of Corti (white dashed box). The right panel shows a higher-magnification view of the organ of Corti. Prominent TRPV4 immunoreactivity was observed beneath IHCs (red arrowhead) and in OHCs (pink arrowheads). **B** In whole-mount preparations, TRPV4 expression was detected beneath IHCs in the synaptic region and in OHCs, including the stereocilia (blue arrowhead). **C** In the SV, the TRPV4 signal showed strong co-localization with Kir4.1-positive intermediate cells. **D** In the SGN, TRPV4 immunoreactivity was predominantly associated with TUJ1-positive neuronal elements. **E** Representative western blots showing TRPV4 expression at 3 h, 1 day, and 3 days after KM/FS administration. **F** TRPV4 expression in the cochlea was upregulated 3 h after KM/FS administration (*n* = 2 per group). β-actin was used as a loading control.　FS furosemide, IHC inner hair cell, KM kanamycin, OHC outer hair cell, SGN spiral ganglion neuron, SV stria vascularis.

TRPV4 affects intracochlear dynamics in AG [13]. Next, TRPV4 expression dynamics in naïve mouse cochleae were examined using TRPV4 immunostaining. Cross-sectional immunostaining, consistent with previous reports [14], demonstrated TRPV4 expression in the organ of Corti, SV, and SGNs (Fig. 2A). In the organ of Corti, higher-magnification analyses revealed prominent TRPV4 immunoreactivity beneath IHCs and along a portion of the OHC plasma membrane near Deiters’ cell phalanges. Surface preparations further confirmed TRPV4 expression in OHCs and their stereocilia (Fig. 2B). Consistent with the cross-sectional findings, strong TRPV4 expression was also observed in the synaptic region beneath IHCs, partially overlapping with the synaptic markers CtBP2 and GluR2, suggesting partial localization near cochlear synaptic regions. In the SV, marker-based co-immunostaining demonstrated that TRPV4 signal strongly co-localized with Kir4.1-positive intermediate cells (Fig. 2C). In the SGNs, TRPV4 immunoreactivity was predominantly associated with TUJ1-positive neuronal elements, indicating that TRPV4 is mainly localized to SGN neuronal structures (Fig. 2D). KM administration altered intracochlear TRPV4 expression [15]. Temporal changes in cochlear TRPV4 expression following intraperitoneal KM and/or FS administration were quantitatively evaluated via western blotting (Fig. 2E and SI Appendix, Fig. S1). Concurrent KM/FS administration caused an acute elevation in TRPV4 expression 3 h post-administration, followed by a gradual reduction to baseline expression levels within 1–3 days (Fig. 2F). In contrast, administration of KM or FS alone resulted in a marginal increase in TRPV4 expression with minimal alterations compared with combined KM/FS administration. Cross-sectional TRPV4 immunostaining 3 h post-KM/FS administration revealed no marked changes in expression sites compared with naïve mice (SI Appendix, Fig. S2).

### Effects of systemic administration of TRPV4 agonist/antagonist on the inner ear

To examine the involvement of TRPV4 in KM-induced ototoxicity, TRPV4 antagonists and agonists were administered intraperitoneally, and pathophysiology was assessed. Initially, to assess the potential toxicity of intraperitoneally administered selective TRPV4 antagonists (HC-067047) [16] or selective agonists (GSK1016790A*)* [17, 18], cochlear function and histology were examined without KM administration (SI Appendix, Fig. S3A). ABR examination conducted 1 week after 3 days of intraperitoneal administration of a TRPV4 antagonist, agonist, or vehicles indicated no significant changes in hearing thresholds in any group (two-way ANOVA, *p* = 0.09, SI Appendix, Fig. S3B). One week post-administration, cochleae were extracted for histological analysis of HCs, cochlear ribbon synapses, and SGNs. No marked HC loss was observed in any group (SI Appendix, Fig. S3C). Quantitative assessment demonstrated nearly 100% OHC and IHC survival in both groups, with no significant differences (two-way ANOVA, OHC; *p* = 0.43, IHC; *p* = 0.97, SI Appendix, Fig. S3D). Furthermore, SGN survival assessed by H&E staining of cochlear cross-sections at the middle turn showed no significant decrease (two-way ANOVA, *p* = 0.10, SI Appendix, Fig. S3E). These findings indicate that TRPV4 antagonists, agonists, and vehicle controls can be administered safely for three consecutive days without affecting cochlear histophysiology.

### Therapeutic effect of the TRPV4 agonist/antagonist on KM/FS-induced ototoxicity

In the next phase of the study, TRPV4 antagonists and agonists were administered to a KM/FS-induced ototoxicity murine model to evaluate hearing thresholds and cochlear pathology. Initially, to investigate the effects of TRPV4 antagonists or agonists on FS, mice were co-administered FS with either a TRPV4 antagonist or agonist, and changes in auditory thresholds were monitored over time. Consistent with prior reports [19], FS temporarily elevated auditory thresholds at 1 h post-administration, followed by a trend toward recovery to baseline levels within ~24 h. FS-treated mice receiving TRPV4 antagonists or agonists exhibited ABR thresholds comparable to those of the FS vehicle group (two-way ANOVA, *p* = 0.40, SI Appendix, Fig. S4). This finding indicates that TRPV4 agonists and antagonists do not have a synergistic effect with FS in elevating ABR thresholds.

**Fig. 3:**
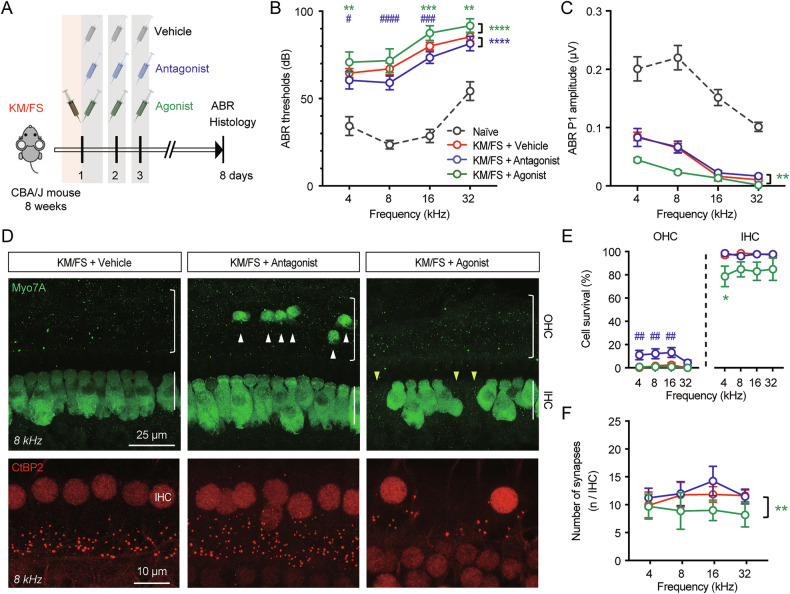
Effects of TRPV4 agonists/antagonists on KM-induced ototoxicity. **A** Experimental protocol. **B** ABR thresholds 1 week after KM/FS administration in combination with TRPV4 agonist/antagonist treatment. ABR hearing thresholds significantly increased in the TRPV4 agonist group compared with those in the vehicle group, whereas the antagonist group exhibited significantly lower hearing thresholds than in the vehicle group. *n* = 6–10 per group. **C** Quantitative assessment of ABR peak 1 (P1) amplitude in each group. ABR P1 amplitude was significantly reduced in all groups compared with that in the naïve group. ABR P1 amplitude significantly decreased in the agonist group relative to that in the vehicle group. *n* = 6–10 per group. **D** Representative immunostaining of OHCs (white bracket) and IHCs (white line) with Myo7A and synaptic ribbons with CtBP2, 1 week after KM/FS administration in combination with TRPV4 agonists/antagonists. In the antagonist group, OHCs exhibited patchy survival (white arrowheads), whereas in the agonist group, OHC loss and IHC patchy degeneration were observed (yellow arrowheads). **E** Quantification of OHC and IHC survival 1 week after KM/FS administration. A significant increase in OHC survival was observed in the antagonist group and a significant decrease in IHC survival was observed in the agonist group. *n* = 5 per animal group. **F** Quantification of CtBP2-positive synaptic ribbon 1 week after KM/FS administration. A significant reduction in the number of synaptic ribbons was observed in agonist-treated groups. *n* = 5 per animal group. Asterisks and hash signs indicate significant differences between the KM/FS + Agonist and KM/FS + Vehicle groups, and between the KM/FS + Antagonist and KM/FS + Vehicle groups, respectively (two-way ANOVA followed by the Dunn’s post hoc test). *, ^#^*p* < 0.05, **, ^##^*p* < 0.01, ***, ^###^*p* < 0.001, ^####^*p* < 0.0001. ABR auditory brainstem response, FS furosemide, IHC inner hair cell, KM kanamycin, OHC outer hair cell.

Subsequently, in the KM-induced ototoxicity model, a single dose of KM/FS was administered on day 1, followed by intraperitoneal administration of TRPV4 antagonists or agonists for three consecutive days. Hearing thresholds and cochlear pathology were assessed after 1 week (Fig. 3A). A body weight reduction of 5–10% was observed in all groups on day 2, followed by gradual recovery. After 1 week, all groups gained weight relative to the naïve group, with no significant differences among groups throughout the experimental period (two-way ANOVA mixed-effects, *p* = 0.15, SI Appendix, Fig. S5A). ABR hearing thresholds were elevated in all groups relative to pre-administration levels (Fig. 3B and SI Appendix, Fig. S5B). Notably, ABR hearing thresholds in the TRPV4 agonist group were significantly higher than those in the vehicle group, whereas the antagonist group exhibited significantly lower hearing thresholds than the vehicle group (two-way ANOVA: KM/FS + vehicle vs. KM/FS + agonist, *p* < 0.0001; KM/FS + vehicle vs. KM/FS + antagonist, *p* < 0.0001). Additionally, ABR peak 1 (P1) amplitudes, reflecting summed neural responses primarily from auditory neurons, were significantly reduced in both TRPV4 antagonist and agonist groups compared with the naïve group. Specifically, P1 amplitude in the agonist group markedly decreased compared with that in the vehicle group (two-way ANOVA, KM/FS + vehicle vs. KM/FS + agonist, *p* = 0.002, Fig. 3C), indicating that severe cochlear degeneration may have occurred in the agonist group. Cochlear histopathology using Myo7A staining was used to assess HC survival (Fig. 3D). KM/FS-induced ototoxicity caused substantial OHC loss, whereas IHC survival was preserved. In the antagonist group, OHCs exhibited patchy survival with nearly complete IHC preservation, whereas in the agonist group, substantial OHC loss and patchy IHC deterioration were observed. Quantitative HC analysis showed significantly increased OHC survival in the antagonist group and significantly reduced IHC survival in the agonist group (two-way ANOVA, OHC: KM/FS + vehicle vs. KM/FS + antagonist, *p* < 0.0001; IHC: KM/FS + vehicle vs. KM/FS + agonist, *p* < 0.0001; Fig. 3E). Furthermore, CtBP2 staining revealed a significant reduction in the number of ribbon synapses in the agonist group (two-way ANOVA, KM/FS + vehicle vs. KM/FS + agonist, *p* < 0.008; Fig. 3F), whereas a substantial proportion remained intact in the vehicle and antagonist groups. Cross-sectional evaluation showed no histological differences in SV or SGN at the cochlear middle turn among the TRPV4 antagonist, agonist, and vehicle groups (one-way ANOVA, SV thickness, *p* = 0.21; SGN, *p* = 0.97; SI Appendix, Fig. S5C–F).

### Evaluation of texas red-labeled gentamicin (GTTR) cochlear uptake and apoptosis

**Fig. 4:**
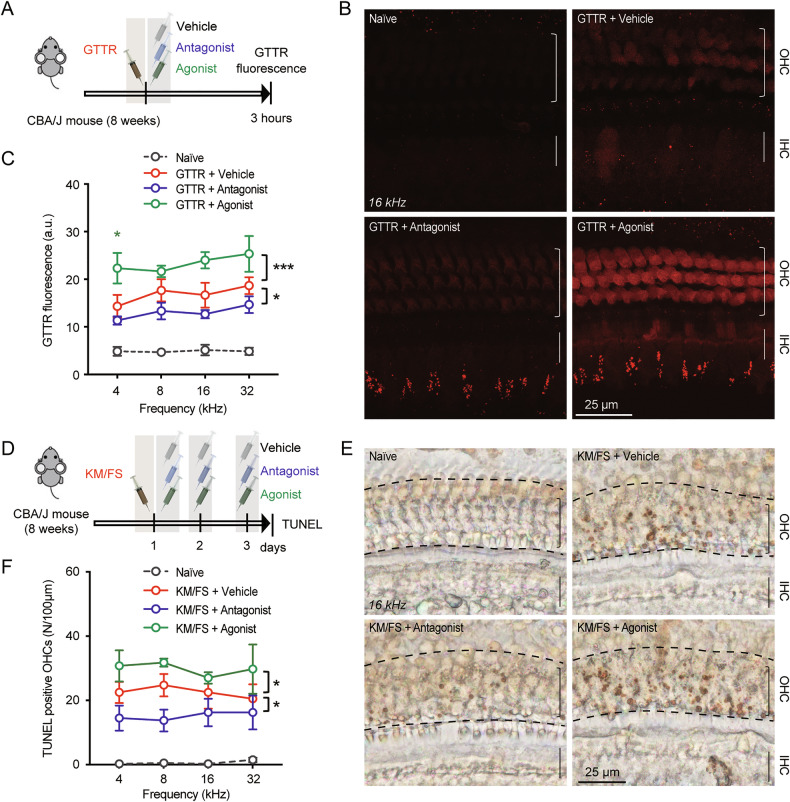
GTTR uptake and KM-induced apoptosis. **A** Experimental protocol for GTTR cochlear uptake evaluation. **B** Representative images of GTTR fluorescence in HCs 3 h after GTTR administration. **C** Quantitative evaluation of GTTR fluorescence. Fluorescence intensity was significantly elevated in the agonist-treated group and decreased in the antagonist-treated group. *n* = 3 per animal group. Asterisks indicate significant differences between the KM/FS + Agonist and KM/FS + Vehicle (two-way ANOVA followed by Dunn’s post hoc test). **D** Experimental protocol for TUNEL staining. **E** Representative photomicrographs of TUNEL staining in HCs 3 days after KM/FS administration. Black dotted lines indicate the OHC region. **F** Quantitative evaluation of the number of TUNEL-positive HCs. TUNEL-positive HCs significantly decreased in the antagonist group and significantly increased in the agonist group relative to the vehicle group. *n* = 4 per animal group. Asterisks indicate statistically significant differences. *p < 0.05, ***p < 0.001. FS furosemide, IHC inner hair cell, KM kanamycin, OHC outer hair cell.

This study demonstrated that KM-induced ototoxicity was mitigated by a TRPV4 antagonist and exacerbated by agonist treatment. To elucidate the underlying pathological mechanism, Texas Red-labeled gentamicin (GTTR; AAT Bioquest), a fluorescent AG conjugate dye, was used to monitor cochlear dynamics (Fig. 4A). GTTR (2 mg/kg, intraperitoneal) was administered, and cochleae were removed 3 h later to assess intracochlear GTTR distribution via fluorescence microscopy (Fig. 4B). Texas Red fluorescence was not detected in the naive group that did not receive GTTR. In the GTTR-treated group, GTTR was incorporated into HCs. Quantitative analysis of GTTR fluorescence intensity in cochlear HCs revealed a significant reduction in the antagonist group, whereas a significant increase was observed in the agonist group compared with the vehicle group (two-way ANOVA, KM/FS + vehicle vs. KM/FS + antagonist, *p* = 0.04, KM/FS + vehicle vs. KM/FS + agonist, *p* = 0.0002, Fig. 4C). The fluorescence intensity trend was consistent across all frequency regions. In non-HC regions, cross-sectional analysis showed GTTR expression, with a significant increase in SV fluorescence intensity after GTTR treatment (two-way ANOVA, *p* = 0.02); however, no marked change was observed in groups treated with TRPV4 antagonists or agonists (two-way ANOVA, KM/FS + vehicle vs. KM/FS + antagonist vs. KM/FS + agonist, *p* = 0.99; SI Appendix, Fig. S6A, B). In addition, no significant change in fluorescence intensity was observed in SGNs at the cochlear middle turn 3 h after GTTR administration (two-way ANOVA, *p* = 0.92).

To elucidate the mechanism of HC death via AG uptake, apoptosis was assessed using TUNEL staining. No TUNEL-positive cells were detected in either OHC or IHC in the naïve group without KM/FS treatment (Fig. 4D). In the vehicle group, abundant OHCs and few IHCs were TUNEL-positive. Quantitative analysis showed a significant decrease in TUNEL-positive HCs in the antagonist group and a significant increase in the agonist group relative to the vehicle group (two-way ANOVA, KM/FS + vehicle vs. KM/FS + antagonist, *p* = 0.04*;* KM/FS + vehicle vs. KM/FS + agonist, *p* = 0.04; Fig. 4F). These findings suggest that intraperitoneally administered KM penetrates the cochlea, enters HCs, and induces apoptotic HC death. Conversely, agonists promote KM influx into the cochlea, enhancing apoptosis.

### Potentiation of ototoxicity via TRPV4 agonist in combination with KM

**Fig. 5:**
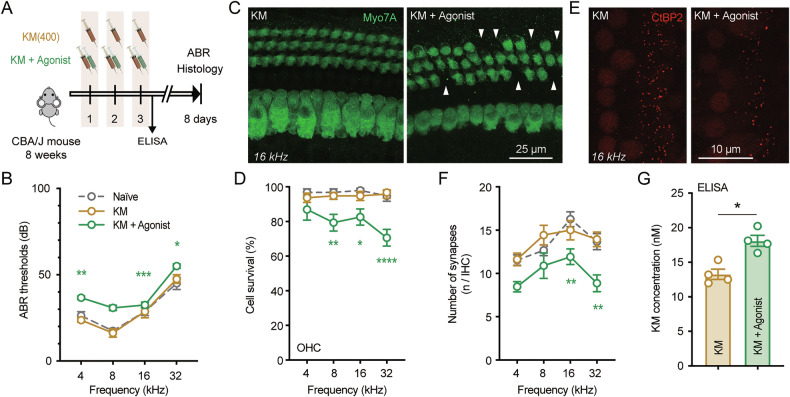
Effect of TRPV4 agonists on KM-induced ototoxicity without FS. **A** Experimental protocol for evaluating the effects of the TRPV4 agonist on KM-induced ototoxicity. **B** ABR thresholds 1 week after KM administration. Mice treated with KM + Agonist showed significantly higher ABR thresholds than those treated with KM alone. *n* = 4 per animal group. **C** Representative immunostaining of HCs with Myo7A 1 week after KM administration. Sparse OHC loss is observed in the KM + Agonist group (white arrowheads). **D** Quantification of OHC survival 1 week after KM administration. The OHC survival rate in KM + Agonist mice was significantly lower than that in KM mice alone. *n* = 4 per animal group. **E** Representative immunostaining of synaptic ribbons with CtBP2 1 week after KM administration. **F** CtBP2-positive synaptic ribbon quantification 1 week after KM administration. The number of synaptic puncta significantly decreased in KM + Agonist mice. *n* = 4 per animal group. **G** ELISA quantification of cochlear KM concentrations 3 days after KM administration. The KM concentration of KM + Agonist was significantly higher than that in the KM alone group. *n* = 4 per animal group. Asterisks indicate statistically significant differences between the KM + Agonist and naïve mice, respectively (two-way ANOVA followed by Dunn’s post hoc test). *p < 0.05, **p < 0.01, ***p < 0.001, ****p < 0.0001. ABR auditory brainstem response, FS furosemide, IHC inner hair cell, KM kanamycin, OHC outer hair cell.

In a complementary experiment examining the effect of a TRPV4 agonist on KM intracochlear influx, auditory and histological assessments were conducted 1 week after administration of KM alone or in combination with a TRPV4 agonist (Fig. 5A). Administration of KM alone for three consecutive days did not affect hearing thresholds, whereas combined administration of KM and TRPV4 agonists caused a significant elevation of hearing thresholds (two-way ANOVA, KM vs. naïve, *p* = 0.98, KM + agonist vs. naïve, *p* < 0.0001, Fig. 5B). One week after administration, cochleae were removed, and surviving HCs were quantified via Myo7A staining. Sparse OHC loss was observed in the KM + agonist group (Fig. 5C). Subsequent quantitative analysis of surviving OHCs demonstrated significantly reduced OHC survival in the agonist group, consistent with the hearing threshold results (two-way ANOVA, KM + agonist vs. naïve, *p* < 0.0001, Fig. 5D). Additionally, CtBP2 staining revealed a marked reduction in the number of ribbon synapses in the agonist group (Fig. 5E). Quantitative analysis of ribbon synapse counts demonstrated a significant decrease across all frequency regions in the agonist group (two-way ANOVA, KM + agonist vs. naïve, *p* < 0.0001; Fig. 5F). Furthermore, cochlear KM concentrations were measured on the third day of treatment using ELISA, revealing a significant increase in the KM + agonist group (Mann–Whitney, *p* = 0.029, Fig. 5G). These findings suggest that TRPV4 agonists enhance KM uptake in the cochlea, promoting KM-induced ototoxicity.

### Evaluation of GTTR uptake and HC survival via cochlear explant

**Fig. 6:**
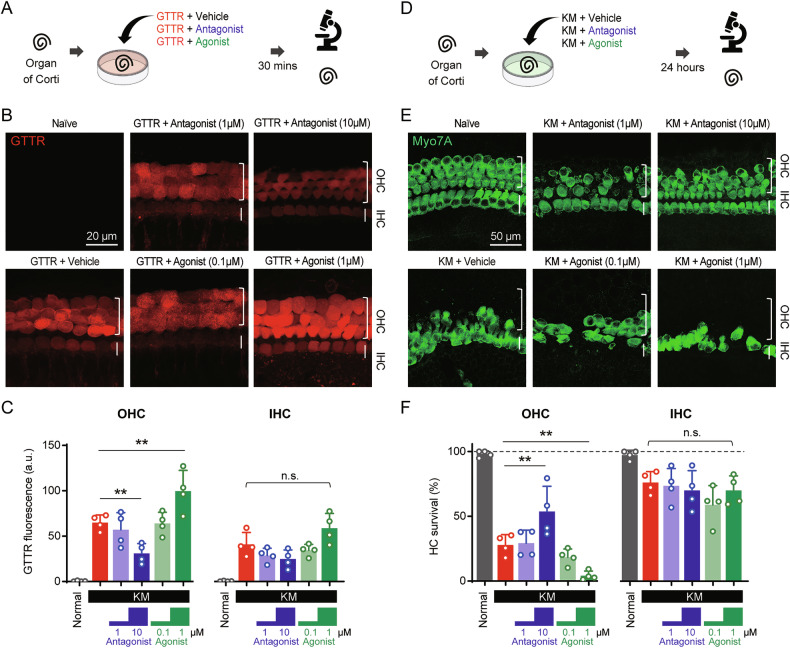
Effects of TRPV4 agonists and antagonists on GTTR uptake and HC survival in cochlear explants. **A** Experimental protocol for GTTR staining in cochlear explants. **B** Representative images of GTTR fluorescence intensity in OHCs (white bracket) and IHCs (white line). **C** Quantitative analysis of GTTR fluorescence intensity in HCs. In OHCs, low concentrations of the TRPV4 antagonist (1 µM) and agonist (0.1 µM) caused no marked changes in GTTR uptake, whereas the antagonist (10 µM) significantly decreased GTTR uptake and the agonist (1 µM) significantly increased GTTR uptake compared with the vehicle group. No marked changes were observed in IHCs. *n* = 4 per group. **D** Experimental protocol for HC survival analysis following KM exposure in cochlear explants. **E** Representative images of HCs immunostained for Myo7A. **F** Quantitative analysis of OHC survival 24 h after KM exposure. Low concentrations of the TRPV4 antagonist (1 µM) and agonist (0.1 µM) caused no marked changes in OHC survival, whereas the antagonist (10 µM) significantly increased OHC survival and the agonist (1 µM) significantly decreased OHC survival compared with the vehicle group. OHC loss was more pronounced than IHC loss. *n* = 4 per group. Asterisks indicate statistically significant differences. **p < 0.01, ****p < 0.0001. IHC inner hair cell, KM kanamycin, OHC outer hair cell.

Whether TRPV4 antagonists or agonists modulate cochlear KM influx from the SV into the endolymph, HC uptake of KM from the endolymph, or both remains unclear. TRPV4 expression has been reported to increase in a spatiotemporal developmental pattern from embryonic day 17 (E17) to postnatal day 6 (P6) in mice [20]. In this study, we used organ-of-Corti explant cultures from P4 mouse pups to investigate the mechanisms underlying HC uptake of KM. We then examined the effects of TRPV4 antagonists and agonists on GTTR uptake by HCs (Fig. 6A). Initially, we confirmed TRPV4 expression in HCs in the P4 mouse organ culture (SI Appendix, Fig. S6), consistent with previous reports [6]. Next, GTTR was incubated with either a TRPV4 antagonist or agonist for 30 min, and strong GTTR fluorescence was observed in HCs compared with the naïve group (Fig. 6B). Notably, GTTR uptake in OHCs was greater than that in IHCs. Co-incubation with a TRPV4 antagonist (1 or 10 µM) or agonist (0.1 or 1 µM) revealed concentration-dependent effects on GTTR uptake in OHCs. Low concentrations of the antagonist (1 µM) and agonist (0.1 µM) produced no marked changes in GTTR uptake, whereas a higher concentration of the antagonist (10 µM) reduced GTTR uptake, and a higher concentration of the agonist (1 µM) increased GTTR uptake (Fig. 6B). In contrast, no substantial changes in GTTR uptake were observed in IHCs. Quantitative evaluation of GTTR fluorescence intensity confirmed that, in OHCs, GTTR uptake was significantly decreased in the antagonist (10 µM) group and significantly increased in the agonist (1 µM) group compared with the vehicle group (one-way ANOVA, OHC: vehicle vs. antagonist [10 µM], *p* < 0.01; vehicle vs. agonist [1 µM], *p* < 0.01, Fig. 6C).

KM was subsequently applied to cochlear explants to investigate the effects of antagonists and agonists on KM-induced HC death (Fig. 6D). After 24 h of incubation, the naïve group without KM showed no HC loss, whereas KM exposure caused substantial HC loss, with OHC loss being more pronounced than IHC loss. Consistent with the GTTR uptake results, low concentrations of the TRPV4 antagonist (1 µM) and agonist (0.1 µM) produced no marked changes in OHC survival (Fig. 6E). In contrast, co-incubation with a higher concentration of the antagonist (10 µM) increased OHC survival, whereas a higher concentration of the agonist (1 µM) decreased OHC survival. Quantitative evaluation of OHC survival confirmed a significant increase in the antagonist (10 µM) group and a significant decrease in the agonist (1 µM) group compared with the vehicle group (one-way ANOVA, OHC: vehicle vs. antagonist [10 µM], *p* < 0.01; vehicle vs. agonist [1 µM], *p* < 0.01; Fig. 6F). These findings suggest that TRPV4 contributes to intracochlear KM dynamics by regulating both SV permeability and HC KM uptake.

### Effect of TRPV4 agonist/antagonist on KM antimicrobial activity

**Fig. 7:**
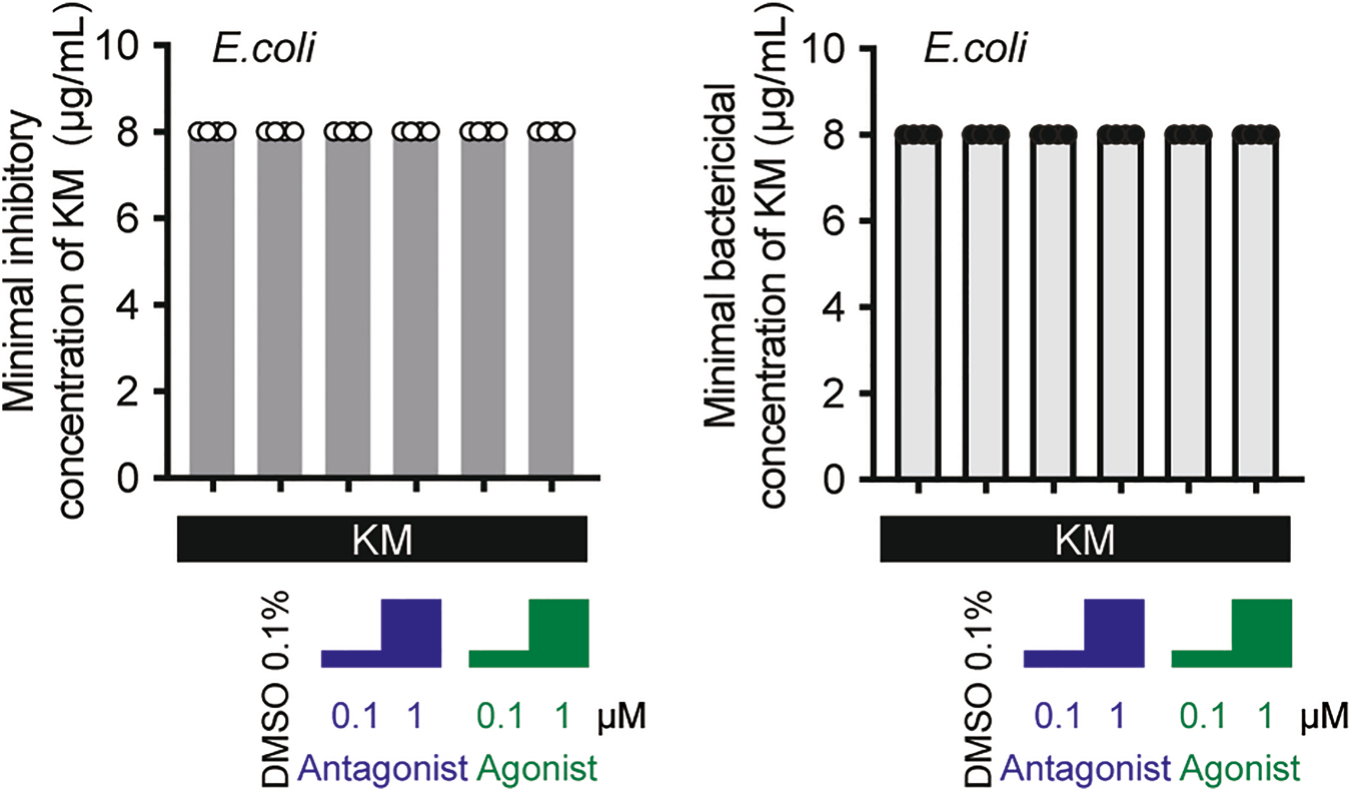
TRPV4 agonists/antagonists had no effect on KM’s antimicrobial activity against *E*. *coli.* MICs and MBCs of KM with various TRPV4 agonist/antagonist concentrations were quantified. KM robustly inhibited the growth of and killed *E*. *coli* cells at 8 µg/mL, regardless of the presence of TRPV4 agonists or antagonists. *n* = 4 for each group. KM kanamycin, MIC minimum inhibitory concentration, MBC minimum bactericidal concentration.

Finally, we examined the effects of TRPV4 agonists and antagonists on KM antimicrobial activity against *E. coli* in vitro (Fig. 7). KM exhibited minimal inhibitory and bactericidal concentrations of 8 µg/mL regardless of TRPV4 modulation. Collectively, these results suggest that TRPV4 modulation can alter cochlear influx of KM while preserving its antimicrobial efficacy.

## Discussion

AG-induced cochlear ototoxicity remains a major clinical challenge. Despite their essential role against multidrug-resistant infections, AGs cause irreversible ototoxicity, posing a global health concern [3]. Hearing loss in children hinders communication, education, and social integration, leading to lifelong economic and psychosocial consequences [21]. Despite decades of research, no clinical intervention has successfully separated AG bactericidal effects from ototoxicity. This study identifies TRPV4 as a key mediator of AG transport and ototoxicity and shows that its pharmacological modulation preserves antibacterial activity. AGs accumulate in cochlear HCs and SGNs, causing ROS generation, mitochondrial dysfunction, calcium imbalance, and apoptosis [22]. However, intracochlear AG dynamics remain unclear, particularly how AGs cross the blood–labyrinth barrier (BLB) to reach cochlear targets. Evidence shows that: (i) fluorescently labeled AGs demonstrate entry into the endolymph and accumulation in HCs [23]; (ii) MET channels act as pathways for cationic AGs, and MET inhibition reduces AG uptake and HC damage [24]; and (iii) the TRP family contributes to cochlear susceptibility and AG uptake under inflammatory conditions [1, 7, 25]. However, these findings alone do not fully explain AG intracochlear dynamics or provide clinical therapeutic strategies. Our findings show that TRPV4 functions as an integrated regulatory mechanism. TRPV4 is a Ca^2+^-permeable, mechanosensitive, and osmosensitive cation channel expressed throughout the cochlea [26]. This distribution suggests TRPV4 influences BLB permeability and HC entry [27, 28]. After AG administration, the compound crosses the BLB. TRPV4 expression in SV intermediate cells and vascular endothelium regulates BLB permeability and osmotic stress [29]. Our data show that TRPV4 activation after AG exposure increases BLB permeability, while TRPV4 inhibition reduces AG penetration, preserving auditory thresholds and HC viability.

After entering the cochlear fluid, AG accumulates in sensory HCs, particularly OHCs, which are susceptible to calcium overload [30]. TRPV4, a calcium-permeable channel, facilitates calcium influx and ROS production, potentially causing mitochondrial dysfunction and apoptosis [31]. Our in vivo and cochlear explant data suggest that TRPV4 contributes to intracochlear AG dynamics at multiple levels. In vivo, TRPV4 modulation altered cochlear AG accumulation and ototoxic outcomes, supporting a role for TRPV4 in regulating drug entry across the BLB, particularly at the level of the stria vascularis. Importantly, the explant experiments further showed that, under higher concentrations of selective TRPV4 modulators, TRPV4 agonists and antagonists modestly altered GTTR uptake and HC survival, indicating that TRPV4 also contributes directly to AG permeation at the HC membrane. Thus, TRPV4 appears to function as a modulatory component of AG trafficking both during systemic-to-endolymph transport and during subsequent HC uptake. This interpretation also helps reconcile our findings with previous reports. Although earlier studies primarily used broad-spectrum TRP channel inhibitors [6], our study employed selective TRPV4 agonists and antagonists, which may account for differences in the observed effects on GTTR uptake. In addition, differences in developmental stage, species, GTTR dose, and tissue composition between experimental systems may further influence the contribution of TRPV4 to cochlear drug entry. Given that AG can permeate the cochlea through multiple partially redundant pathways, TRPV4 is unlikely to be the sole determinant of drug entry; rather, it likely acts as one of several modulatory routes that together shape cochlear AG load and vulnerability to ototoxic injury.

This framework also helps explain why the observed quantitative effects of TRPV4 modulation were modest. In our in vivo paradigm, ototoxic injury was intentionally induced under highly severe conditions to ensure robust and reproducible cochlear damage. Under such conditions, the apparent protective effect of TRPV4 antagonism or the aggravating effect of TRPV4 agonism may be underestimated because cochlear injury approaches a ceiling, and parallel AG-entry pathways are likely to dominate when exposure is extreme. Thus, the dynamic range for detecting larger pharmacological effects may have been compressed in the present model, and more moderate injury paradigms may be better suited to quantify the full magnitude of TRPV4-dependent protection or sensitization. Importantly, even modest shifts may still be physiologically meaningful in a threshold-dependent toxicity setting, in which relatively small differences in cochlear AG burden or cellular stress may determine whether vulnerable HCs and synapses remain below or exceed the threshold for irreversible damage.

AG administration downregulates TRPV4 expression in the inner ear [15]. Conversely, TRPV4 expression increased after KM/FS administration. This may be due to variations in dosage, subtype, species, and timing of AG administration. TRPV4 regulation is biphasic, showing upregulation during initial stress followed by desensitization [32, 33]. Our findings show that toxicity increased with selective TRPV4 agonist administration and decreased with antagonist treatment, suggesting that initial upregulation contributes to cochlear vulnerability rather than reflecting stress alone. Clinically, these findings suggest a novel therapeutic strategy. Several otoprotective agents reduce AGHL; however, some approaches face challenges, including limited inner ear specificity or systemic effects [34–36]. TRPV4 inhibition preserved auditory function while maintaining KM antibacterial activity. Several TRPV4 antagonists have advanced to early clinical development for pulmonary and vascular conditions, with safety data available [37, 38]. This supports repurposing TRPV4 antagonists for otoprotection in AG therapy, particularly relevant in resource-constrained regions where AGs remain primary therapeutic agents.

This study has limitations. First, although TRPV4 was strongly implicated in AG transport and ototoxicity, the precise molecular mechanisms remain unclear. Mechanisms include direct translocation via TRPV4, modulation of channel gating, or indirect regulation of barrier permeability, requiring electrophysiological and structural studies.

An additional limitation is that our in vivo model was designed to produce robust ototoxicity, which may have reduced the dynamic range for detecting larger pharmacological effects of TRPV4 modulation. Therefore, future studies using more moderate injury paradigms may be important for more precisely defining the quantitative contribution of TRPV4 to cochlear AG trafficking and protection.

Second, this study was conducted in mice and given the differences in cochlear structure between mice and humans, validation in higher mammals and clinical trials are needed. Third, as this study focused on KM, future research should explore other AGs, such as gentamicin and amikacin, to establish generalizability. Finally, given TRPV4’s roles in osmosensation, vascular tone, and epithelial barrier function, its long-term inhibition requires careful safety evaluation. Since TRPV4 is osmosensitive, if FS disrupts cochlear osmotic balance, changes in TRPV4 expression or activation would not be unexpected. Therefore, cochlear effects of FS should be assessed with caution. Detailed investigations are needed to clarify how FS co-administration affects systemic and serum KM concentrations, as such changes could influence cochlear drug uptake and toxicity.

In conclusion, this study identified TRPV4 as a viable target for mitigating AG-induced ototoxicity. TRPV4 inhibition decouples antibacterial efficacy from sensory toxicity, offering a promising clinical solution to AG therapy challenges. Combined with previous research on MET and TRP pathways, these findings enhance the understanding of cochlear vulnerability mechanisms and support further research for safer antibiotic use. Given the global reliance on AGs where alternatives are limited, TRPV4 inhibition holds substantial potential to save lives while preserving hearing.

## Materials and methods

### Animals

All animal experiments were performed in accordance with the guidelines of the Animal Experimentation and Ethics Committee of Kitasato University School of Medicine. The animals were maintained at room temperature (RT) of 24 ± 1 °C under a 14 h:10 h light:dark cycle. All mice used in this study were age-matched male CBA/J mice at 8 weeks of age.

### ABR

ABR measurements were performed as described previously [39]. Briefly, the mice were anesthetized using an intraperitoneal injection of midazolam (4 mg/kg), medetomidine (0.75 mg/kg), and butorphanol (5 mg/kg). Measurements were conducted in a soundproof room, where two silver-coated screws (diameter: 1 mm) were inserted at the midline of the vertex of anesthetized mice (one on the forehead as the negative electrode and the other as the positive electrode). The ground electrode was then inserted subcutaneously into the back. Tone burst stimuli with a 1-ms rise/fall time at frequencies of 4, 8, 16, and 32 kHz were generated. A total of 256 responses were band-pass filtered at 50–3000 Hz and averaged using a NeuroPack Sigma system (Nihon Kohden). ABR waveforms were recorded at 5 dB sound pressure level (SPL) intervals, starting from 80 dB stimulation and decreasing until no waveform could be visualized. ABR threshold was defined as the lowest stimulus intensity that produced a reliable peak I or IV. The amplitude of peak I, which reflects cochlear neural activity, was measured.

### Cochlear immunohistochemistry

Immunohistochemical studies were performed as previously described [40]. Mice were intracardially perfused with 4% paraformaldehyde (PFA) in phosphate buffer (PB). The cochleae were removed, and fixative flow was facilitated by opening the round and oval windows and perforating the bone over the apex. The tissues were post-fixed in 4% PFA in PB for 1 h and decalcified in 5% ethylenediaminetetraacetic acid (EDTA) for 16 h at 4 °C to facilitate dissection. Subsequently, cochlear tissues were dissected into microsegments of the auditory epithelium. Whole-mount sections were blocked in 5% normal goat serum (Vector Laboratories) with 0.3% Triton X-100 (Wako) in phosphate-buffered saline (PBS) for 1 h. Subsequently, primary antibodies were added, followed by rinsing and incubation with the secondary antibody. Anti-GluR2 (mouse IgG2a; Millipore), anti-CtBP2 (mouse IgG1; BD Transduction Laboratories), anti-myosin7a (rabbit IgG; Proteus Biosciences), anti-TRPV4 (rabbit; Alomone Labs), and anti-NF-κB (Chichen; Millipore) were used as the primary antibodies. The tissues were incubated with GluR2/CtBP2 for 20 h at 4 °C, followed by two consecutive 1-h incubations at RT with appropriate Alexa Fluor (1:500 in blocking buffer; Molecular Probes). The organ of Corti was mounted with an antifade mounting medium (VECTASHIELD; Vector Laboratories) and examined using confocal laser microscopy (LSM710, Zeiss).

### Assessment of HC survival and CtBP2 expression

HCs and synapses stained with Myo7A and CtBP2/GluR2 were viewed using a confocal laser microscope (LSM710, Zeiss). We used a custom-written ImageJ plugin (https://myfiles.meei.harvard.edu/xythoswfs/webui/_xy-e693768_1-t_wC4oKeBD). Furthermore, we computed a cochlear frequency map to precisely localize the inner HCs (IHCs) from the 4-, 8-, 16, and 32 kHz regions. For HC assessment, we calculated the ratio of surviving HCs as a percentage. For synaptic assessment, we obtained confocal z-stack images of the 4-, 8-, 16, and 32 kHz regions in the IHC area of each ear using a high-resolution, oil immersion objective (×63) with 2× digital zoom and confocal laser microscopy with a 0.25-µm step size. The z-stacks spanned all IHCs to include all synaptic specializations within the image. We imported the image stacks into ImageJ software. Subsequently, CtBP2 puncta in each image stack were automatically captured and counted. The number of synaptic ribbons per IHC within a 100 μm range was counted in each of the four frequency-specific regions.

### Assessment of SGN density and SV thickness

To quantitatively assess the number of SGNs, decalcified cochleae were embedded in Tissue-Tek O.C.T (Sakura) and sectioned at a thickness of 5 µm near Rosenthal’s canal. The sections were stained with hematoxylin and eosin (H&E) and viewed under a light microscope (Olympus BX53 microscope with an Olympus DP27 camera). For SGN density measurement, we counted the number of SGNs in the middle turn of Rosenthal’s canal and calculated the SGN density per 10,000 μm^2^. SV thickness, from the endolymphatic surface of the marginal cells to the spiral ligament side of the basal cells, was measured at the thickest portion of the SV, and the average SV thickness was calculated.

### ELISA

Cochlear KM concentrations were determined using a KM ELISA kit (MET-5144, CELL BIOLABS INC) according to the manufacturer’s protocol. For cochlear samples, whole cochleae were used. Intracochlear KM concentrations were calculated by comparing samples with standard curves generated using reagents provided in the kit.

### GTTR

For the in vivo study, GTTR (purchased from AAT Bioquest) was administered intraperitoneally (2 mg/kg) to mice with TRPV4 agonists or antagonists 3 h before cardiac perfusion. Cochleae were removed, and GTTR fluorescence was observed via confocal fluorescence microscopy (LSM980, Zeiss). GTTR fluorescent pixel intensities were obtained using the histogram function of ImageJ software (Fiji, National Institutes of Health). Mean intensity values normalized to the control standard across multiple experimental sets.

For cochlear explants, cochleae from 3- to 5-day-old postnatal C57BL/6 mice were dissected in Hanks’ solution (Gibco). To obtain a flat cochlear surface, the spiral ganglion, Reissner’s membrane, and most of the basal cochlear segment were removed. Explants were plated onto 4-well plates (Greiner Bio-One) coated with poly-L-ornithine (0.1 mg/mL, Fujifilm) and laminin (50 µg/mL, Sigma-Aldrich). Cochlear explants were cultured with TRPV4 agonists or antagonists for 5 min, and GTTR was added to a final concentration of 0.2 μM for a further 10 min. All cultures were maintained in a 5% CO_2_/20% O_2_-humidified incubator (MCO-5AC-PJ, Panasonic). GTTR fluorescence was observed using a confocal fluorescence microscope (LSM980, Zeiss). The average background fluorescence value was subtracted from each OHC value to obtain the final intensity value for each OHC.

### Terminal deoxynucleotidyl transferase dUTP nick end labeling (TUNEL)

TUNEL staining was performed using a TUNEL Kit (G7362, Promega), according to the manufacturer’s protocol. TUNEL-positive HCs were counted using an Olympus BX53 microscope with an Olympus DP27 camera.

### Western blot

The cochleae were removed and homogenized in RIPA buffer with a protease inhibitor cocktail (Nacalai Tesque). The homogenate was centrifuged at 10,000 rpm for 10 min at 4 °C. The supernatants were separated by sodium dodecyl sulfate–polyacrylamide gel electrophoresis (e-PAGEL-HR, ATTO), and the proteins were transferred onto an Immobilon-P membrane (Clear Blot Membrane-P Plus, ATTO). The membranes were blotted with antibodies against anti-TRPV4 (Alomone Labs, ACC-034, rabbit, 1:400) and β-actin (A5441, mouse, 1:2000, Sigma). The membranes were then treated with secondary antibodies (MBL330 and NA934, 1:5000, GE Healthcare), and the protein bands were visualized using a chemiluminescence detection system (ECL Select Western Blotting Detection Reagent, Amersham). Immunoblot signals were analyzed using an IQ-800 digital imaging system (Cytiva). Band intensities were quantified and normalized to their corresponding loading controls (β-actin).

### Cochlear explants

The cochleae of 4-day-old postnatal C57BL/6 mice were dissected in Hanks’ solution (Gibco). To obtain a flat cochlear surface, the spiral ganglion, Reissner’s membrane, and most of the basal cochlear segment were removed. Explants were plated onto 4-well plates (Greiner Bio-One) coated with poly-L-ornithine (0.1 mg/mL, Fujifilm) and laminin (50 µg/mL, Sigma-Aldrich). Cochlear explants were incubated with KM in the presence of dimethyl sulfoxide (DMSO), TRPV4 agonists, or antagonists in a humidified incubator (MCO-5AC-PJ; Panasonic). Following 24 h incubation, the organs of Corti were fixed for 30 min with 4% PFA in PB. Immunostaining was initiated by blocking tissues for 1 h with 0.1% Triton X-100 in PBS supplemented with 5% goat serum. The fixed and permeabilized tissues were incubated overnight with primary antibodies against Myo7A (Proteus Biosciences). The samples were washed thrice for 20 min. Primary antibodies were detected using secondary antibodies conjugated to Alexa Fluor 488 (Molecular Probes). The samples were viewed using a confocal fluorescence microscope (LSM980, Zeiss).

### Measurement of antimicrobial activity

The antimicrobial activity was measured by NDTS Inc. (Hokkaido, Japan). Briefly, *E. coli* (JCM5491, RIKEN BRC-JCM) was inoculated onto Mueller–Hinton broth (MHB) agar medium in a biosafety cabinet (MHE-S1301A2-PJ, PHC) and incubated at 37 °C for 16–20 h in a heater-type incubator (MIR-262, SANYO). To prepare the inoculum suspension, single colonies formed on LB agar plates were dissolved using a sterile disposable loop in glass test tubes (BSN, 519-BSNBS050102MK100) containing 5 mL of sterile PBS, yielding four bacterial suspensions. The turbidity of the suspensions was adjusted to a McFarland standard of 0.5 (McF 0.5) using a BSN turbidimeter (DEN-600, BS-050109-AAK) and BSN McFarland standard solution (BS-050102-BK). The adjusted suspensions were used as inocula. The lowest antibiotic concentration sufficient to inhibit bacterial growth was defined as the minimum inhibitory concentration (MIC). To determine the minimum bactericidal concentration (MBC) of KM, 50 µL of KM-containing bacteria-inoculated tubes that exhibited no bacterial growth during the MIC assays were streaked onto Müller–Hinton agar and incubated overnight at 37 °C.

### Statistical analysis

Statistical analyses were performed using GraphPad Prism 8.2.1 (GraphPad Software Inc., La Jolla, CA, USA). All data were first checked for normality using the Shapiro–Wilk test. Among-group comparisons were conducted using one-way analysis of variance (ANOVA) with correction for multiple comparisons using Dunn’s post hoc test when the data were normally distributed. Non-normally distributed data were analyzed using the non-parametric Kruskal–Wallis test, followed by Dunn’s multiple comparison test. Statistical differences in ABR examination and synaptic counts were analyzed using two-way ANOVA followed by Dunn’s multiple comparisons test, based on the normality confirmed by the Shapiro–Wilk test. All data are presented as the mean ± standard error. All data were derived from independent biological samples, and sample sizes were selected empirically without prespecified effect size assumptions. The experiments were performed by a third party who was unaware of the allocation of samples and animals to the different experimental groups. The statistical significance level was set at *p* < 0.05. *p* values are indicated as *, ^#^*p* < 0.05, **, ^##^*p* < 0.01, ***, ^###^*p* < 0.001, ****, ^####^*p* < 0.0001.

## Supplementary information

Supplemental material

## Data Availability

The data that support the findings of this study are openly available within the article and/or supplementary materials.
