# Supplementary material for: TRPV4 mediates aminoglycoside trafficking and ototoxicity without compromising antimicrobial efficacy
Source: Cell Death Discov. 2026 Apr 25;12:272. doi: 10.1038/s41420-026-03132-9 (PMC13243603; doi:10.1038/s41420-026-03132-9)
Supplement: Supplementary file 1 — Supplemental material [file 41420_2026_3132_MOESM1_ESM.docx]

**TRPV4 mediates aminoglycoside trafficking and ototoxicity without compromising antimicrobial efficacy**

Runnung Title: TRPV4 Modulates Aminoglycoside Trafficking and Cochlear Toxicity

Lingshuai Kong,^a,b^ Takaomi Kurioka,^a,c^* Sachiyo Mogi,^a^ Yoshihiro Nitta,^a^ Kengo Yamamoto,^a^ and Taku Yamashita^a^

**Supporting Information Figures**

**
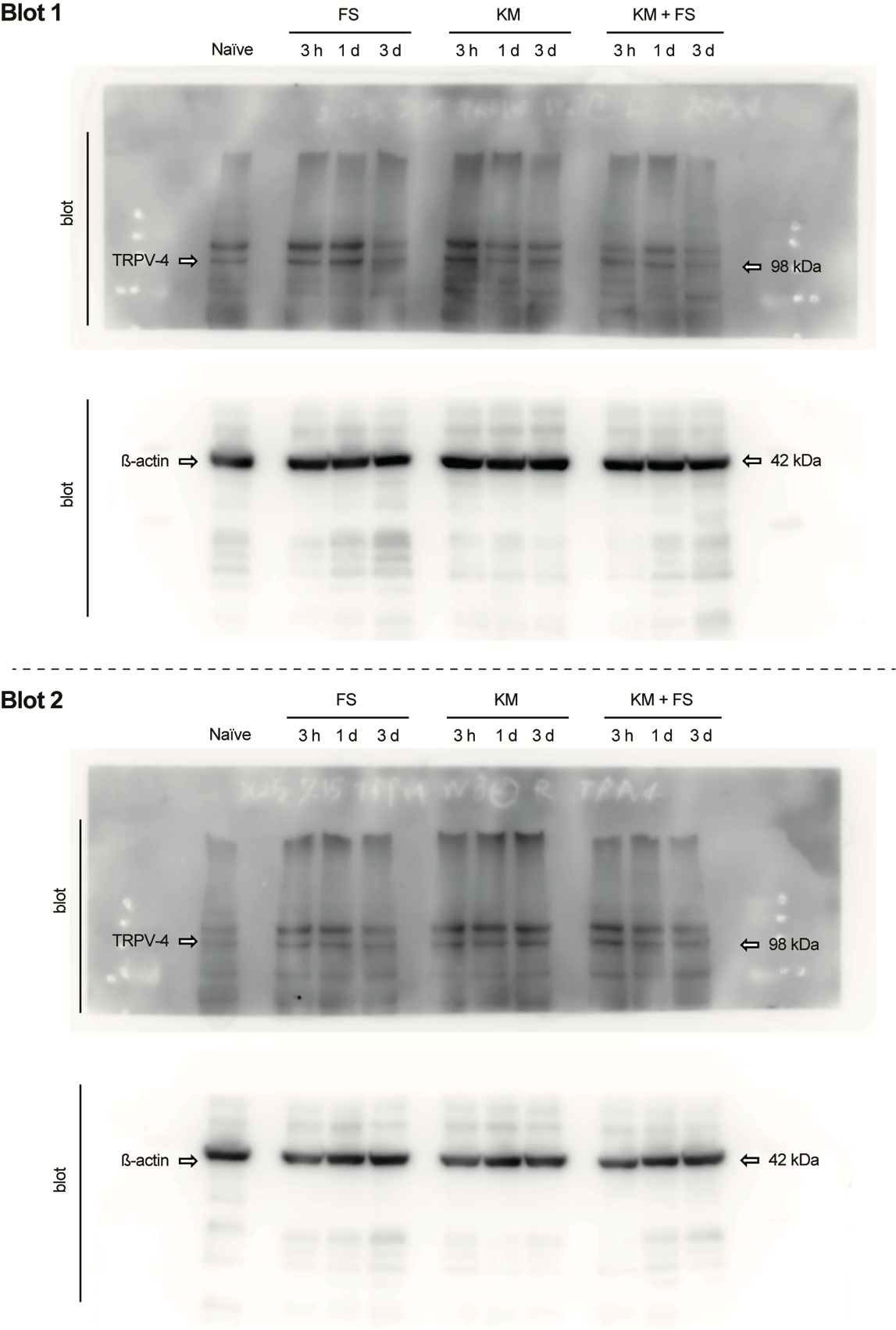
**

**Figure S1.** Western Blot Raw Data for TRPV4.


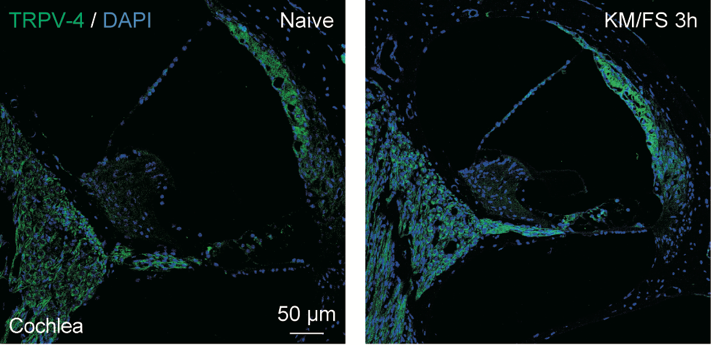


**Figure S2.** Representative micrographs of cochlear sections immunostained for TRPV4 in naïve mice and in mice at 3 h after KM/FS administration.

**
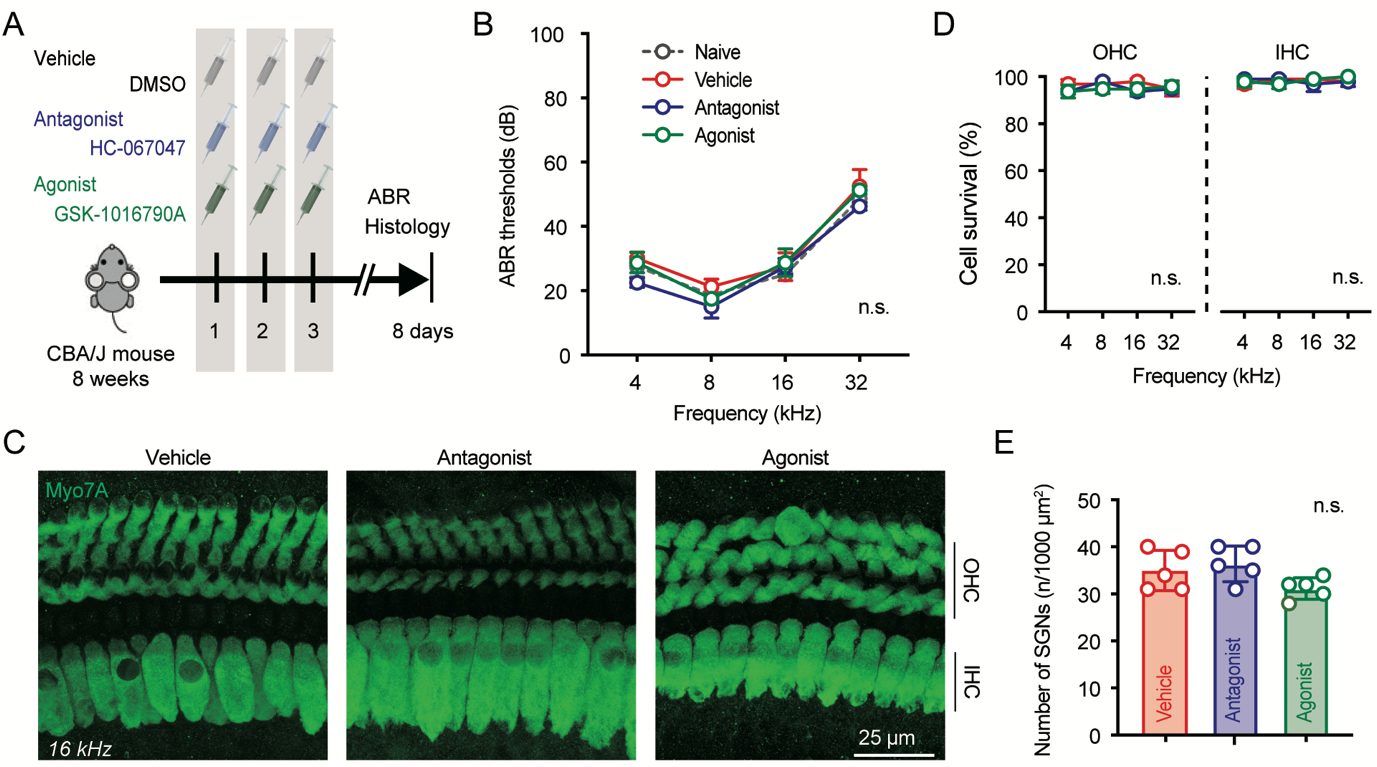
**

**Figure S3.** Effects of TRPV4 agonists/antagonists on KM-induced ototoxicity. (*A*) Experimental protocol for evaluating the effects of TRPV4 agonist/antagonist on normal mouse ears. (*B*) ABR thresholds one week after three consecutive days of TRPV4 agonist/antagonist administration. No significant differences in ABR hearing thresholds were observed between groups. n = 5 per animal group. (*C*) Representative immunostaining of HCs for Myo7A. None of the mice exhibited apparent HCs loss. (*D*) Quantification of HC survival revealed no significant loss of OHC or IHC staining in any group. n = 5 per animal group. (*E*) Quantitative assessment of SGN density shows no significant differences among the groups. n = 5 per animal group. ABR, auditory brainstem response; IHC, inner hair cell; OHC, outer hair cell.


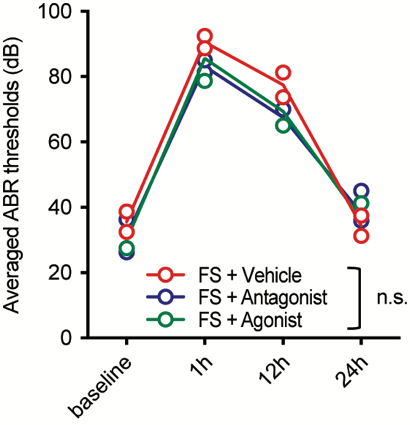


**Figure S4.** Hearing thresholds following FS administration in combination with a TRPV4 agonist/antagonist. FS temporarily elevated ABR thresholds within 1h post-administration, with a tendency toward recovery to baseline levels within approximately 24 h. The FS combined with TRPV4 agonist/antagonist treatment group exhibited ABR threshold changes that were comparable to those of the FS vehicle group. n = 2 per animal group. ABR, auditory brainstem response; FS, furosemide.


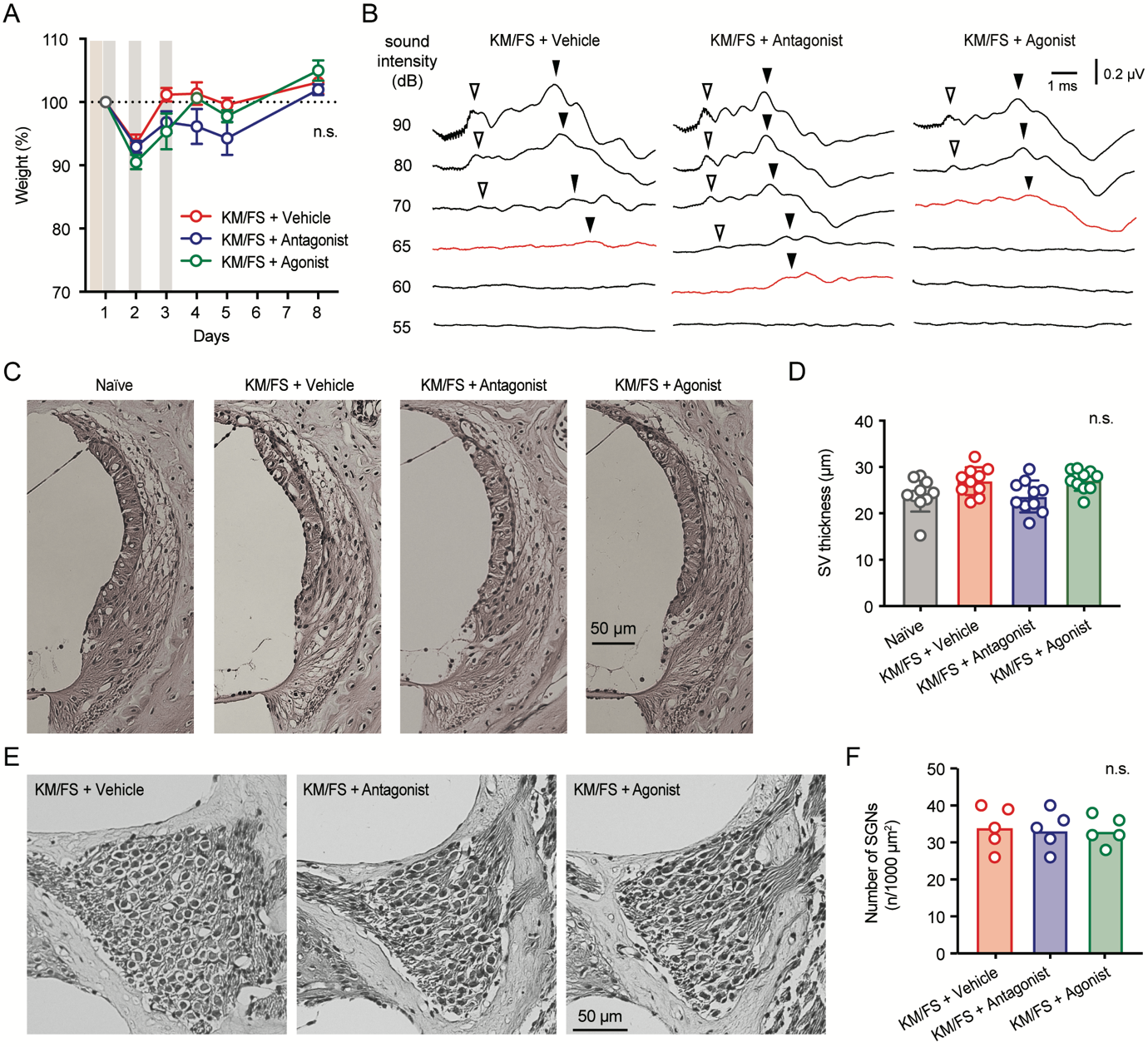


**Figure S5.** Body weight, ABR waveform, and cochlear pathology following KM-induced toxicity in combination with TRPV4 agonist/antagonist. (*A*) Body weight changes (%) following KM/FS administration combined with a TRPV4 agonist/antagonist. n = 5 per animal group. (*B*) Representative ABR waveforms for each group at one week after KM/FS administration. White and black arrowheads indicate ABR P1 and P5, respectively. (*C*) Representative photomicrographs of the SV at the basal turn*.* (*D*) Quantitative assessment of SV thickness demonstrated no significant differences among the groups of mice. (*E*) SGNs were densely packed in all the groups. n = 10 per animal group. (*F*) Quantitative assessment of SGN density showed no significant differences between the groups of mice. n = 5 per animal group. ABR, auditory brainstem response; FS, furosemide; KM, kanamycin; SGN, spiral ganglion neuron; SV, stria vascularis.


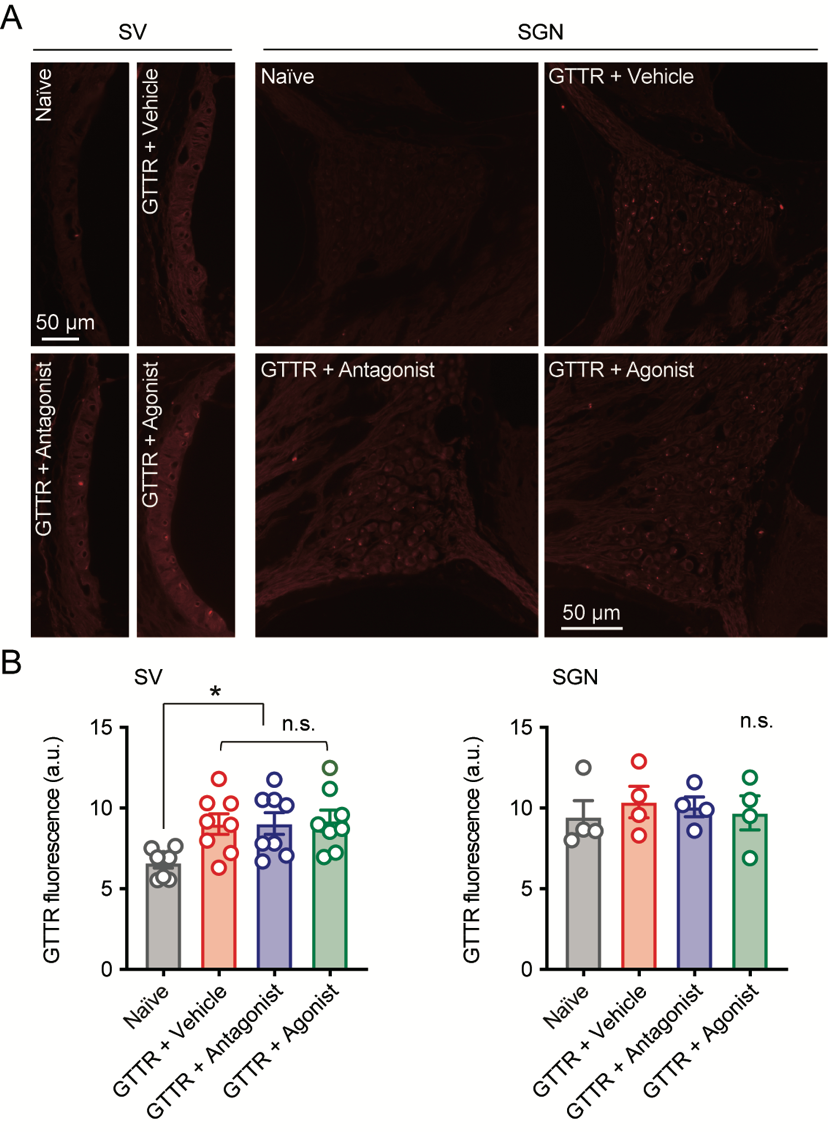


**Figure S6.** GTTR uptake in SV and SGN following GTTR administration in combination with an agonist/antagonist. (*A*) Representative images of GTTR fluorescence in SV and SGN. (*B*) Quantitative evaluation of GTTR fluorescence intensity. There was a stronger uptake of GTTR in the SV regions in the GTTR treatment groups than in the naïve group. The TRPV4 agonist/antagonist did not significantly affect GTTR uptake fluorescence at either SV or SGN. n = 4 for each animal group for SGN assessment and n = 8 for each animal group for SV assessment. SGN, spiral ganglion neuron; SV, stria vascularis.


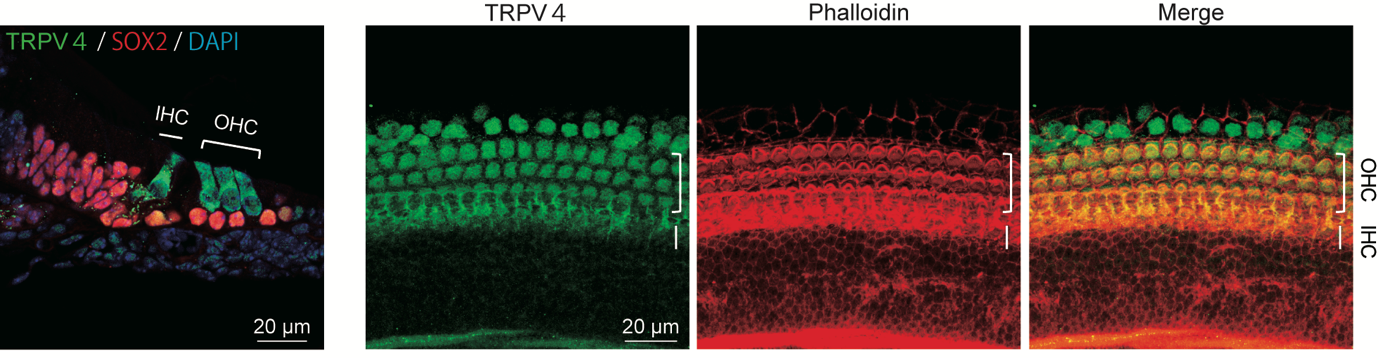


**Figure S7.** Representative micrographs of P4 cochlea stained for TRPV4. TRPV4 expression was detected in IHCs and OHCs. IHC, inner hair cell; OHC, outer hair cell.
